# Cytoarchitectonic Maps of the Human Metathalamus in 3D Space

**DOI:** 10.3389/fnana.2022.837485

**Published:** 2022-03-08

**Authors:** Kai Kiwitz, Andrea Brandstetter, Christian Schiffer, Sebastian Bludau, Hartmut Mohlberg, Mona Omidyeganeh, Philippe Massicotte, Katrin Amunts

**Affiliations:** ^1^Cécile and Oskar Vogt Institute of Brain Research, University Hospital Düsseldorf, Medical Faculty, Heinrich Heine University, Düsseldorf, Germany; ^2^Max Planck School of Cognition, Stephanstraße 1a, Leipzig, Germany; ^3^Institute of Neuroscience and Medicine (INM-1), Forschungszentrum Jülich, Jülich, Germany; ^4^Helmholtz AI, Forschungszentrum Jülich, Jülich, Germany; ^5^McGill Centre for Integrative Neuroscience, McConnell Brain Imaging Center, Montreal Neurological Institute, McGill University, Montreal, QC, Canada; ^6^National Research Council of Canada, Ottawa, ON, Canada

**Keywords:** metathalamus, BigBrain, cytoarchitectonic maps, lateral geniculate body, medial geniculate body, human, 3D histology

## Abstract

The human metathalamus plays an important role in processing visual and auditory information. Understanding its layers and subdivisions is important to gain insights in its function as a subcortical relay station and involvement in various pathologies. Yet, detailed histological references of the microanatomy in 3D space are still missing. We therefore aim at providing cytoarchitectonic maps of the medial geniculate body (MGB) and its subdivisions in the BigBrain – a high-resolution 3D-reconstructed histological model of the human brain, as well as probabilistic cytoarchitectonic maps of the MGB and lateral geniculate body (LGB). Therefore, histological sections of ten postmortem brains were studied. Three MGB subdivisions (MGBv, MGBd, MGBm) were identified on every 5th BigBrain section, and a deep-learning based tool was applied to map them on every remaining section. The maps were 3D-reconstructed to show the shape and extent of the MGB and its subdivisions with cellular precision. The LGB and MGB were additionally identified in nine other postmortem brains. Probabilistic cytoarchitectonic maps in the MNI “Colin27” and MNI ICBM152 reference spaces were computed which reveal an overall low interindividual variability in topography and extent. The probabilistic maps were included into the Julich-Brain atlas, and are freely available. They can be linked to other 3D data of human brain organization and serve as an anatomical reference for diagnostic, prognostic and therapeutic neuroimaging studies of healthy brains and patients. Furthermore, the high-resolution MGB BigBrain maps provide a basis for data integration, brain modeling and simulation to bridge the larger scale involvement of thalamocortical and local subcortical circuits.

## Introduction

The human metathalamus, located caudoventrally of the main body of the thalamus, plays an important role in processing visual and auditory information. Visual and auditory processing is encoded separately in the two major nuclei of the metathalamus, i.e., the lateral geniculate body (LGB) and the medial geniculate body (MGB). The LGB is a 6-layered structure, innervated by optic tract fibers covering the contralateral visual field. Its two magnocellular and four parvocellular layers process functionally distinct retinal pathways. The MGB on the other hand receives input from ascending tonotopically organized projections *via* the medial lemniscus, as well as projections from the inferior colliculus and the auditory cortex (Peruzzi et al., 1997; Saint Marie et al., 1997; Llano and Sherman, 2008; Caspary and Llano, 2017). It can cytoarchitectonically be subdivided into three major compartments: the ventral, dorsal and medial subdivisions (Winer, 1984). Both nuclei have prominent projections to cortical areas and serve as subcortical relay stations.

Investigating the structural-functional relationship of the MGB and LGB including its subdivisions and layers is also relevant from a clinical perspective, e.g., to understand the MGB’s involvement in tinnitus (Llinas et al., 2002; Rauschecker et al., 2010; Leaver et al., 2011; Ridder et al., 2015; Caspary and Llano, 2017; Berlot et al., 2020), speech recognition (Mihai et al., 2019), and developmental dyslexia (Díaz et al., 2012) as well as the LGB’s role in glaucoma (Wang et al., 2015; Stein et al., 2021), multiple sclerosis (Korsholm et al., 2007; Papadopoulou et al., 2019), Parkinson’s (Lee et al., 2016), and psychiatric diseases (Butler and Javitt, 2005; Selemon and Begovic, 2007).

Since the spatial resolution of ultra-highfield fMRI has increased to the submillimeter range in recent years, more detailed studies have been become feasible including the possibility to measure laminar brain activity (Huber et al., 2018; Jia et al., 2021) as well as identifying functional subdivisions of subcortical (Rijk et al., 2021) and cortical (Martino et al., 2015; Nasr et al., 2016) structures.

However, existing histological maps of the human thalamus do not include subdivisions of the MGB and/or layers of the LGB or do not cover the metathalamus over its whole extent (Morel, 2007; Krauth et al., 2010; Ding et al., 2016; Mai et al., 2016). The same holds true for MRI based probabilistic atlases of the thalamus (Iglesias et al., 2018; Najdenovska et al., 2018; García-Gomar et al., 2019). Furthermore, no probabilistic histologically based reference maps of the metathalamus exist so far, which make it difficult to account for individual variability in topography and volume, as well as to compare histological maps with findings from neuroimaging. More detailed maps of subdivisions and layers of the MGB and LGB could provide micro-anatomical reference data for high-field MRI investigations, to inform neuroimaging studies, and to provide reference data for biologically realistic brain modeling and simulation.

The BigBrain model based on its 7404 cell-body stained and 3D-reconstructed sections constitutes an anatomical brain model at a spatial resolution of 20 micrometers isotropic in this regard (Amunts et al., 2013). It has been used, for example, to interpret MRI based models of brain connectivity (Wei et al., 2019; Paquola et al., 2020b), functional and structural gradients (Paquola et al., 2019; Royer et al., 2020), as well as default mode network components (Margulies et al., 2016; Paquola et al., 2019).

In the present study, we aimed to create a cytoarchitectonic map of the MGB and its subdivisions in the BigBrain model and supplement previously published maps of the LGB with its six layers (Brandstetter et al., 2021). To construct a high-resolution map of the MGB, a novel deep-learning based cytoarchitectonic mapping tool was applied (Schiffer et al., 2021c). Secondly, the MGB and LGB were identified in histological sections of ten postmortem brains and volumes, as well as probabilistic cytoarchitectonic maps were computed to address the intersubject variability of the two nuclei.

## Materials and Methods

### Processing of Postmortem Brains

Cytoarchitectonic mapping was performed in serial histological sections of ten human brains from body donors (5 female, age 59–85 years, 5 male, 30–75 years, Table 1). The brains were obtained in accordance to legal and ethical regulations and guidelines as part of the body donor program of the Department of Anatomy of the Heinrich Heine University Düsseldorf. Body donors gave written informed consent for the general use of brain tissue for aims of research and education. All usage in this work is covered by a vote of the ethics committee of the Medical Faculty of the Heinrich Heine University Düsseldorf (#4863). The postmortem delay did not exceed 24–36 h. The list of brains also included the BigBrain dataset (Amunts et al., 2013).

**TABLE 1:** List of postmortem brains used for cytoarchitectonic mapping and analysis.

Brain ID	Gender	Age (Years)	Cause of death	Fresh weight (g)
pm 1	Female	79	Carcinoma of the bladder	1,350
pm 4	Male	75	Necrotizing glomerulonephritis	1,349
pm 5	Female	59	Cardiorespiratory insufficiency	1,142
pm 7	Male	37	Acute right heart failure/cardiac arrest	1,437
pm 8	Female	72	Renal failure/renal arrest	1,216
pm 9	Female	79	Cardiorespiratory insufficiency	1,110
pm 10	Female	85	Mesenteric infarction	1,046
pm 13	Male	39	Drowning	1,234
pm 20	Male	65	Cardiorespiratory insufficiency	1,392
pm 21	Male	30	Bronchopneumonia	1,409

The procedure of processing the brain tissue was described in detail in Amunts et al. (2020). In short, the brains were fixed in 4% buffered formalin (pH 7.4) or Bodian’s fixative for at least 3 months. All brains underwent magnetic resonance imaging using a T1-weighted 3D FLASH sequence (flip angle 40°, repetition time TR 40 ms, echo time TE 5 ms). MR images were used as an undistorted spatial reference for the 3D-reconstruction of the histological sections. After scanning, the brains were embedded in paraffin and serially sectioned in the coronal plane on a large-scale microtome (20 μm thickness), whereby series of blockface images of the paraffin-embedded brains were obtained. Every 15th section (every section in case of the BigBrain) was stained for cell bodies using a silver staining technique (Merker, 1983), and digitized using tissue scanners (1 μm in-plane resolution).

### Cytoarchitectonic Probability Maps

To create probability maps of the MGB and LGB, both nuclei were delineated and traced over their whole extent on every 15*^th^* section (distance between sections: 300 μm) in all 10 brains using an in-house software (Online Section Tracer). The MGB was identified based on previous microscopical studies and its characteristic topography (Kuhlenbeck, 1954; Winer, 1984). Delineation criteria for the LGB were adapted from the literature (Clark, 1932; Brandstetter et al., 2021). The delineations of the MGB and LGB in the left and the right hemisphere were 3D-reconstructed. Hereby, spatial transformations of the whole-brain histological datasets were used that were earlier computed based on the MR-images and the blockface images of the paraffin-embedded brains. The delineated nuclei were then spatially normalized and transferred to the T1-weighted single-subject template of the Montreal Neurological Institute (MNI), “Colin27”, as well as the MNI ICBM152 2009c non-linear asymmetric reference space (Evans et al., 2012). The individual maps of the MGB and the LGB were superimposed in both templates to calculate probabilistic maps. Values from 0 to 100% overlap were calculated to indicate the probability for each voxel of the reference brain to contain either the MGB or the LGB at a certain position (Amunts et al., 2020).

### Volumetric Analysis

Volumes were calculated and corrected for shrinkage based on the delineations of the MGB and LGB in histological sections based on Cavalieri’s principle (Amunts et al., 2007). A volume normalization was applied by calculating the proportion of the volume of the structures and the total brain volume to make the results comparable (Bludau et al., 2014). Differences in volume proportions were tested for significant effects caused by hemisphere (left vs. right) and sex (male vs. female) with pair-wise permutation tests. For each of these tests, the corresponding values (male/female; left/right hemisphere) were grouped and a contrast estimate was calculated between the means of these groups. The null distribution was estimated by a Monte-Carlo simulation. All values were randomly redistributed into two groups, calculating the same contrast with a repetition of 1,000,000 iterations. Differences were considered statistically significant if the contrast estimate of the true comparison was larger than 95% of the values under the null distribution (*p* < 0.05). Differences in mean volumes between the MGB and LGB were analyzed using a paired two-sided *t*-test with an α error-rate set to 0.05.

### High-Resolution Cytoarchitectonic Brain Mapping in the BigBrain

In addition, the MGB and its subdivisions were delineated on every 5th section of the BigBrain dataset (Amunts et al., 2013) using the high-resolution digitized scans. The range of sections covered a distance of 3.20 mm in the left and 3.08 mm in the right hemisphere. To map the three subdivisions on every section, a deep-learning based brain mapping tool designed to map cytoarchitectonic structures in full stacks (Schiffer et al., 2021c) was applied. The deep-learning network architecture has shown to resemble cytoarchitectonic criteria (Kiwitz et al., 2020) and has successfully been used to generate whole-stack maps of several cytoarchitectonic areas (Schiffer et al., 2021c). The method was trained on 57 delineated sections containing the MGB and its subdivisions. Training was performed remotely *via* a web-based interface (Schiffer et al., 2021c) on the supercomputer JURECA at Jülich Supercomputing Centre (Krause and Thörnig, 2018). Automatically created maps were subsequently controlled to exclude falsely qualified sections, which were manually corrected *via* the tool’s web-based interface. The annotations were transformed into the 3D-reconstructed BigBrain space by applying a non-linear registration of the high-resolution digitized sections (Omidyeganeh et al., 2020) and available transformations for the BigBrain (Amunts et al., 2013) to generate a volume for each MGB subdivision. The total number of volume voxels, their physical size and a shrinkage factor of 1.931 for the BigBrain (Bludau et al., 2014) were subsequently used to calculate the volume of the MGB and its subdivisions. The total volume of the subdivisions of this straight-forward approach was compared to the estimated MGB volume based on Cavalieri’s principle as described above.

3D-surface meshes of the subdivisions were generated using the marching cube algorithm (Lewiner et al., 2003). The 3D reconstruction directly followed the protocol described in Schiffer et al. (2021c). Rough edges on the mesh surfaces were subsequently smoothed locally using normalized curvature operators in the normal direction preserving their specific structure. Surface meshes of the LGB have been generated and 3D-reconstructed in a similar manner based on publicly available whole brain maps of the LGB and its layers in the BigBrain (Brandstetter et al., 2021; Schiffer et al., 2021a).

## Results

### Localization of the Medial and Lateral Geniculate Bodies

**FIGURE 1:**
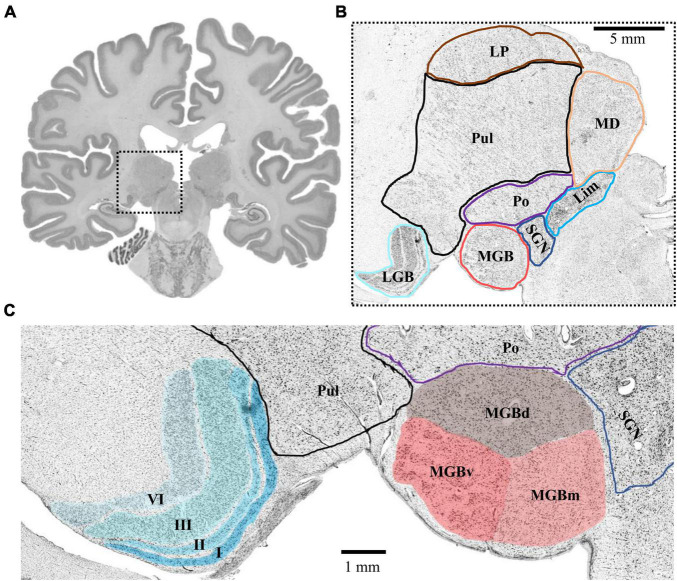
Topography of the MGB and LGB. **(A)** Cell-body stained section (number 3660, caudal MGB) from the left hemisphere of postmortem brain pm 21. The dashed box indicates the location of the thalamus magnified in panel **(B)**. **(B)** Magnified crop from panel **(A)** showing the topography of the MGB and LGB (cyan and red) in comparison to putative locations of other thalamic nuclei. **(C)** Magnified crop from panel **(B)** depicting the layered structure of the LGB (cyan, left side) and the subdivisions of the MGB (red, right side). Roman numerals indicate layers of the LGB; LGB, lateral geniculate body; MGB, medial geniculate body; MGBv, ventral subdivision of the MGB; MGBd, dorsal subdivision; MGBm, medial subdivision; Po, posterior nucleus; SGN, suprageniculate nucleus; Lim, compact limitans nucleus; Pul, pulvinar; MD, mediodorsal nucleus; LP, lateral posterior nucleus.

The MGB and LGB followed a consistent topography in all analyzed postmortem brains. The LGB was located ventrolaterally of the pulvinar of the thalamus. It showed the typical 6-layered pattern with sharp bends. The MGB was always located medially to the LGB. Its caudal pole protruded from the caudal extremity of the diencephalon. The caudal pole itself was located caudoventrally of the posterior nuclear complex (i.e., the compact limitans, suprageniculate and posterior nucleus of the thalamus) and medially of the inferior pulvinar nucleus of the thalamus (Figure 1). The caudal surface of the pretectum formed the dorsomedial flank of the MGB.

### Probabilistic Cytoarchitectonic Maps of the Medial and Lateral Geniculate Bodies

**FIGURE 2:**
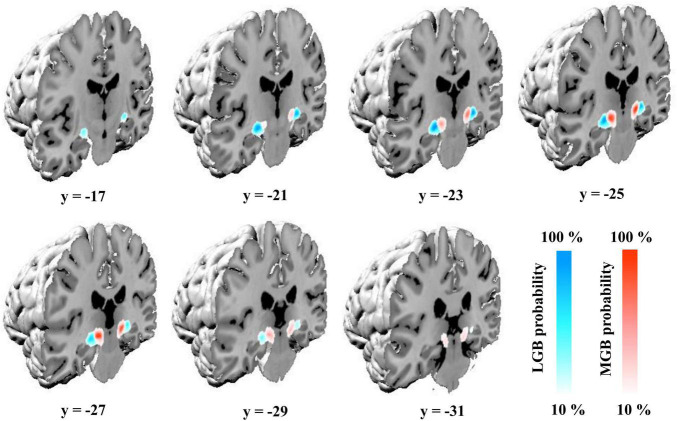
Probability maps of the MGB and LGB in the single-subject template of MNI “Colin27” (Evans et al., 2012). Coronal slices from rostral (*y* = –17) to caudal (*y* = –31) show the probability maps color-coded in blue (LGB) and red (MGB) in the MNI “Colin27” coordinate space. Color gradients on the lower right indicate the overlap across the ten postmortem brains for a specific voxel (10%: only one brain; 100%: all ten brains); LGB, lateral geniculate body; MGB, medial geniculate body.

Delineations of the MGB and LGB in the sample of 10 postmortem brains were transferred to the MNI Colin 27 and MNI ICBM152 2009c non-linear asymmetric reference spaces. The probability maps of the two nuclei show their paired arrangement caudoventrally of the main body of the thalamus. The LGB is located dorsally of the hippocampal formation along its whole extent with the MGB adjoining it medially (Figure 2). Center of mass coordinates in the Colin 27 and MNI ICBM152 2009c non-linear asymmetric spaces (in parentheses) constituted *x* = −24, *y* = −24, *z* = −10 (−24, −25, −9) for he left LGB, *x* = 22, *y* = −24, *z* = −10 (23, −24, −9) for the right LGB, *x* = −16, *y* = −27, *z* = −8 (−15, −27, −7) for the left MGB, as well as *x* = 14, *y* = −27, *z* = −9 (15, −26, −8) for the right MGB. The y-coordinates of the center of masses demonstrate the more rostral location of the LGB (*y* = −24) in comparison to the MGB (*y* = −27) as shown in Figure 2. The color-coded probability maps of both nuclei (Figure 2) show a central peak with a steady decrease when moving away from the center of mass in all three dimensions – emphasizing the central location of the two nuclei within the probability maps in both hemispheres.

### Volumetric Analysis of the Metathalamus

Results of the volumetric analysis of the ten postmortem brains are shown in Table 2. Shrinkage-corrected mean volumes of the MGB (Mean = 258.7 mm^3^, SD = 59.2 mm^3^) were significantly smaller than the LGB (Mean = 332.4 mm^3^, SD = 54.6 mm^3^) volumes (*t*(9) = −7.0, *p* < 0.001, two-sided test). Permutation tests did not reveal any significant effects of hemisphere and sex as well as their interaction on the shrinkage-corrected volumes for each nucleus (*p* > 0.05).

**TABLE 2:** Mean volumes, standard deviations (SD) as well as minimal and maximal values of the shrinkage-corrected mean volumes of the MGB and LGB in ten postmortem brains for both hemispheres measured in mm^3^.

Nucleus	Statistic	Left hemisphere	Right hemisphere	Sum
MGB	Mean	124.3	134.4	258.7
	Min	91.9	89.0	
	Max	164.2	209.3	
	SD	27.4	35.3	59.2
LGB	Mean	166.9	165.5	332.4
	Min	116.3	120.4	
	Max	220.8	218.3	
	SD	28.5	26.6	54.6

*MGB, medial geniculate body; LGB, lateral geniculate body.*

### Cytoarchitecture of the Medial Geniculate Body

**FIGURE 3:**
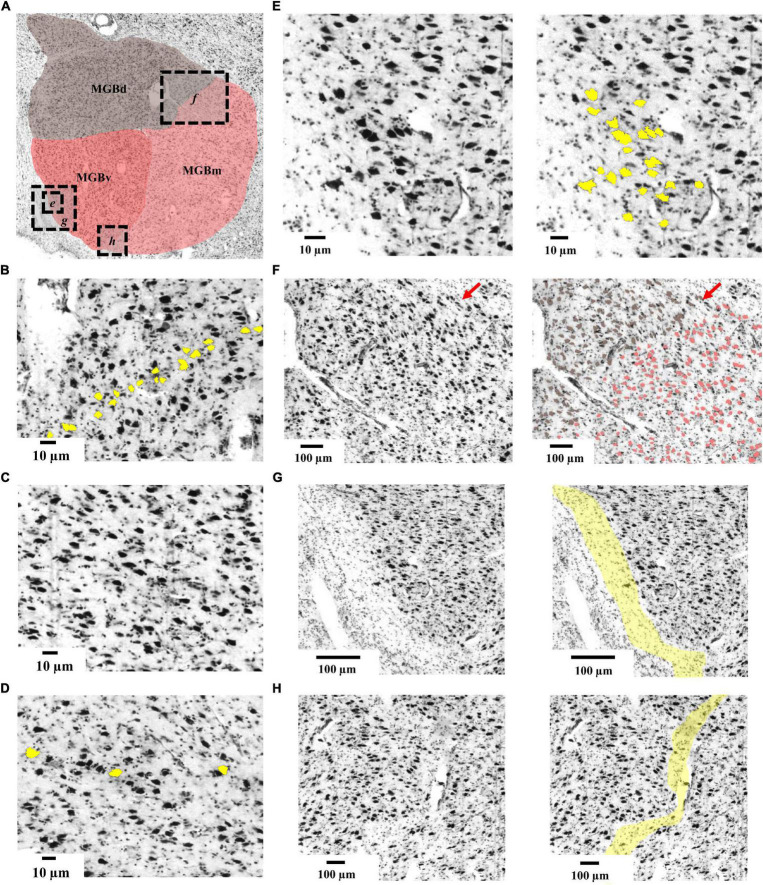
Cytoarchitecture of the MGB. **(A)** Histological cell-body stained section from the left hemisphere of the BigBrain (section 3290, rostral level of the MGB) displaying the three subdivisions. Dashed boxes with italic letters (e-h) indicate magnified excerpts. **(B)** Cytoarchitecture of MGBv showing row-like clusters of medium-sized cells (yellow); **(C)** cytoarchitecture of MGBd showing a cell-sparse pattern. **(D)** Cytoarchitecture of MGBm showing perikarya (yellow) which were larger than in the other subdivisions. **(E)** Magnified excerpts from the ventrolateral quadrant of MGBv showing the putative human homologous region of the feline ventrolateral nucleus (Winer, 1984). **(F)** Magnified excerpts of the border between MGBd and MGBm. Red arrows indicate a cell-sparse zone defining the borderline. Brownish colored cells correspond to the previously described suprageniculate and posterior limitans nuclei of MGBd (Winer, 1984) and are of similar size as the reddish colored cells of MGBm. **(G)** Magnified excerpts from the ventrolateral part of MGBv showing a capsule of neuropil (yellow) flanking MGBv. **(H)** Magnified excerpts of the border between MGBv and MGBm highlighting a cell-sparse zone that defines the borderline; MGB, medial geniculate body; MGBv, ventral subdivision of the MGB; MGBd, dorsal subdivision; MGBm, medial subdivision.

Three subdivisions of the MGB were identified and delineated in the BigBrain (Figure 3): The ventral subdivision (MGBv) formed the ventrolateral part of the MGB and was mainly comprised of small and medium sized perikarya, some of which formed row-like clusters as described previously (Winer, 1984). These contributed to a layer-like appearance of the ventral subdivision (Figure 3B). The MGBv was flanked by white matter that extended ventrally to the lateral border of the cerebral peduncle and ringed the free surface of the caudal pole of the MGB (Figure 3G). It could easily be separated from the medial and dorsal subdivisions by cell-sparse zones (Figure 3H), as well as differences in cell-density, size and composition. Similar to observations by Winer (1984), we found a small cluster of larger cells in the ventrolateral part of the ventral subdivision on some sections (Figure 3E).

The dorsal subdivision (MGBd) covered the whole caudo-rostral extent of the MGB forming a cap on top of the ventral and medial subdivisions. It showed a reduced cell-density in comparison to the ventral subdivision (Figure 3C). The largest cells in the dorsal subdivision could be found on the medial and ventromedial limb, right at the border to the medial subdivision (Figure 3F). They marked the border to the medial subdivision. The border to the medial subdivision was also characterized by a fine cell-sparse zone, which was more profound in rostral sections (Figure 3F).

The medial subdivision (MGBm) formed the ventromedial part of the MGB and, on average, contained the largest perikarya of all subdivisions (Figure 3D). The MGBm showed a caudo-rostral gradient of increasing cell size which facilitated the separation from the ventral subdivision in rostral sections. At the same time, the increase in cell size impeded the separation from the dorsal subdivision with its especially large somata at the border to the medial subdivision (Figure 3F).

### High-Resolution 3D-Reconstructions of the Medial Geniculate Body in the BigBrain

**FIGURE 4:**
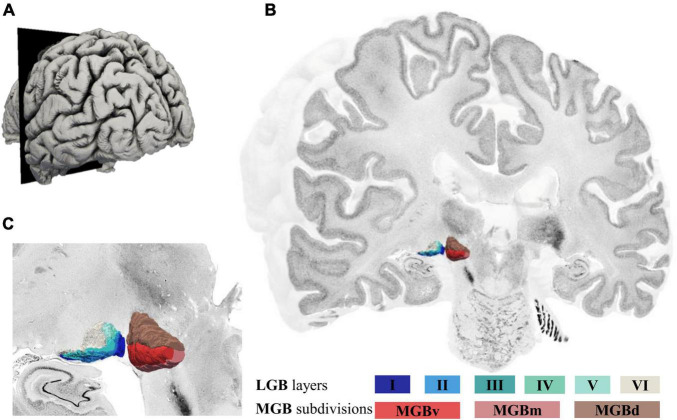
3D-reconstructions of the MGB and LGB in the left hemisphere of the BigBrain. **(A)** Illustration of the BigBrain surface model, latero-caudal view (cutting plane corresponds to coronal section number 3290, rostral MGB). **(B)** Cut through the BigBrain volume at section position 3290 showing the subdivisions of the MGB (MGBv, MGBd, and MGBm) and layers of the LGB (I-VI). **(C)** Magnified view of the LGB (left) and MGB (right) showing their location at the ventral surface of the BigBrain; MGB, medial geniculate body; LGB, lateral geniculate body; MGBv, ventral subdivision of the MGB; MGBd, dorsal subdivision; MGBm, medial subdivision.

The deep-learning based brain mapping tool allowed to identify delineations of the three subdivisions of the MGB on 132 sections of the left and 165 sections of the right hemisphere in the BigBrain. Combined 3D-reconstructions of the MGB and LGB (Brandstetter et al., 2021) with its subdivisions and layers in the BigBrain are shown in Figure 4 (see Supplementary Video) and demonstrate the paired arrangement of the two nuclei in proximity of the hippocampal formation ventrolaterally (Figure 4C). The MGBd forms a cap across the whole extent of the MGB. The ventral and medial subdivisions share the lower half of the MGB. On rostral sections, MGBd and MGBm are flanked by white matter and parts of the ventrobasal complex of the thalamus (Figure 4C). Here, the darkly stained substantia nigra of the mesencephalon can be seen ventromedially of the MGB (Figure 4C). Caudally, the MGBd and MGBm border the posterior nuclear complex along their dorsomedial surface, whereas the MGBd and MGBv border the most caudal tip of the inferior pulvinar nucleus (see Figures 1B,C for an illustration).

Shrinkage-corrected volumes of MGBv and MGBm were larger in the left hemisphere, whereas the MGBd subdivision showed a similar size in both hemispheres (Table 3). The sum of all three subdivisional volume measurements corresponds to the mean MGB volume calculated based on mappings on every 15th section in postmortem brain pm20 (BigBrain). The latter fits within 0.8 standard deviations of the left and 1.3 standard deviations of the right hemisphere of the mean volume measurements based on all ten postmortem brains (Table 2).

**TABLE 3:** Volumes of the MGB subdivisions in the BigBrain for both hemispheres.

	Volume in mm^3^	
	
MGB subdivision	Left hemisphere	Right hemisphere
MGBv	18.7	13.1
MGBd	44.8	45.3
MGBm	39.6	31.6
Sum of subdivisions	103.1	90.0

*Measurements show shrinkage-corrected volumes obtained from the 3D-reconstructed surface meshes of the MGB subdivisions (MGBv, MGBd, and MGBm); MGB, medial geniculate body; MGBv, ventral subdivision of the MGB; MGBd, dorsal subdivision; MGBm, medial subdivision.*

## Discussion

The present study introduces high-resolution 3D brain maps of the human MGB and its subdivisions in the BigBrain utilizing a novel deep-learning based brain mapping tool. Together with the recently published LGB layer maps (Brandstetter et al., 2021) they provide a high-resolution whole-brain histological reference of the metathalamus at 20 micrometer resolution. Additionally, probabilistic cytoarchitectonic maps of the MGB and LGB were calculated in a sample of ten brains, with a spatial resolution of 1 mm. They have been aligned with two commonly used reference spaces (MNI “Colin27” and MNI ICBM152 2009c non-linear asymmetric) and are part the Julich-Brain atlas (Amunts et al., 2020). All datasets are publicly available on EBRAINS (Kiwitz et al., 2021b; Schiffer et al., 2021b) and the multi-level atlas of the Human Brain Project.^1^

### Comparison With Previous Histological Studies and Atlases

The overall characterization of three distinct subdivisions of the MGB in the BigBrain is in accordance to histological studies in human (Hassler, 1959; Winer, 1984; Morel, 2007; Ding et al., 2016; Mai et al., 2016) and animal brains (Morest, 1964; Clerici and Coleman, 1990). The topography of the three MGB subdivisions in the BigBrain resembles that shown by Morel (2007), Hassler (1959), and Winer (1992). Following their localization of the subdivisions, our analysis consolidates the notion for the ventromedial location of the magnocellular subdivision MGBm. This subdivision has previously also been reported to be located more ventrolateral by Amunts et al. (2012). The cytoarchitectonic features in our investigation correspond well with those found by Winer (1984), with the exception of the described size of perikarya in MGBm. In the BigBrain, MGBm still contains the largest perikarya of all subdivisions, yet the size difference seems to be not as distinct as previously described (Winer, 1984). Although we were able to detect some correspondences to even finer subparcellations, i.e., the suprageniculate and posterior limitans nuclei of the dorsal subdivision (Figure 3F) and a cell cluster possibly corresponding to the feline ventrolateral nucleus (Strick and Sterling, 1974; Winer, 1984), further subdivisions found in human (Winer, 1984) and animal studies (Morest, 1964; Harrison and Howe, 1974) could not reliably be replicated in the BigBrain.

### Intersubject Variability of Volumes

Currently available histological atlases mostly contain metathalamic structures based on single brains (Ding et al., 2016) with the exception of Morel (2007), who compared the topography of structures to a previously published atlas using a different postmortem brain. The present analysis addresses intersubject variability in a larger sample. The here provided shrinkage-corrected mean volumes of the MGB add to the limited literature of histological volume measurements (Glendenning and Masterton, 1998; Rademacher et al., 2002; Sitek et al., 2019). The interindividual variability in MGB volume resembles data of a more than twofold variability reported earlier (Rademacher et al., 2002). The same is true for the LGB volumes and their approximately twofold interindividual variation (Zvorykin, 1980; Andrews et al., 1997). Similar to previous histological investigations, we found no significant hemispheric asymmetries of MGB and LGB volumes (Eidelberg and Galaburda, 1982; Andrews et al., 1997; Rademacher et al., 2002).

At the same time, volume measurements derived from MRI-based measurements differ to a varying degree from our histological volumes. Comparable MGB volumes have been reported using postmortem MRI (Sitek et al., 2019) and structural *in vivo* MRI (Kitajima et al., 2015; Amaral et al., 2016). At the same time higher MGB, as well as higher and lower LGB volumes have been reported using functional and structural *in vivo* MR measurements (Li et al., 2012; García-Gomar et al., 2019; Jonak et al., 2020).

These inconsistencies may reflect the inherent difficulty of manually segmenting small subcortical nuclei in MR-images. A direct localization of the LGB in structural MR images under low field strengths (1.5 Tesla) requires prior enhancement and co-registration to anatomical surroundings (Li et al., 2012). At 3 Tesla, the LGB has been delineated indirectly using prior masking (Wang et al., 2015; Cecchetti et al., 2016). A more direct segmentation of the LGB and MGB using structural and diffusion-weighted imaging becomes feasible at higher (7 Tesla) field strengths (García-Gomar et al., 2019). Although the MGB becomes detectable at such field strengths, a clear segmentation has only been reported for postmortem structural MR (Sitek et al., 2019). Yet, even under high field strengths a histological validation still seems to be needed to rule out possible confounds such as low diffusion anisotropy due to crossing fibers, as well as to assist investigators with anatomical landmark information when creating segmentations (García-Gomar et al., 2019). The task difficulty of perceiving the LGB and MGB in MR images is significantly impacted by the image acquisition procedure (Kitajima et al., 2015) – imposing a threat to the objectivity of such segmentations. Therefore, *in vivo* segmentations of metathalamic nuclei in particular for the MGB remains challenging due to the nuclei’s small size and low contrast - confirming the notion for a more precise histologically derived reference that our probability maps provide.

The new maps constitute a probabilistic representation of the MGB and LGB in the general population and include five female and five male donors with a wide age range including brains from older body donors. The older age of some body donors may raise the question of possible structural changes of the MGB in the context of age-related hearing loss. Several studies indicate age-related changes of the subcortical auditory system with regards to neurotransmitter and calcium-binding protein expression (reviewed in Caspary and Llano, 2019). Both for normal aging and pathological conditions such as deafness, structural changes have been reported for the temporal cortex including the primary auditory cortex (Lin et al., 2014; Wong et al., 2014; Qian et al., 2017), but not for the MGB (Stanton and Harrison, 2000; Butler and Lomber, 2013; Caspary and Llano, 2019). Other pathologies like Alzheimer’s disease and Leber’s hereditary disease have shown to alter human MGB volumes (Jonak et al., 2020; Bernstein et al., 2021). The clinical records of the body donors did not include any information of such pathologies, and did not mention any changes in hearing abilities. Therefore, the here presented volumes seem to represent mean volumes of the investigated age range.

### Neuroscientific and Clinical Relevance

The maps are part of the Julich-Brain (Amunts et al., 2020), an atlas that is part of the multilevel atlas of the Human Brain Project and its research infrastructure EBRAINS.^2^ This way, the maps may provide a reference to localize findings from neuroimaging and serve as seed regions for functional connectivity and diffusion weighted imaging analyses. In this regard, they can be used to study brain disorders and functional impairments, including the LGB’s involvement in visual field and eye movement deficits (Dai et al., 2011; Pasu et al., 2015; Usrey and Alitto, 2015; Wang et al., 2015), multiple sclerosis (Sepulcre et al., 2009; Hickman et al., 2014; Papadopoulou et al., 2019), Parkinson’s disease (Lee et al., 2016), psychiatric disorders (Mai et al., 1993; Selemon and Begovic, 2007; Dorph-Petersen et al., 2009), as well as the MGB’s involvement in tinnitus (Llinas et al., 2002; Rauschecker et al., 2010; Leaver et al., 2011; Ridder et al., 2015; Caspary and Llano, 2017; Berlot et al., 2020), and both structures’ involvement in Leber’s hereditary optic neuropathy (Jonak et al., 2020). In tinnitus patients, the maps have the potential to aid future neurosurgical planning for deep-brain stimulation (Smit et al., 2016; van Zwieten et al., 2021). The latter already benefits from the development of multimodal deep-brain stimulation atlases (Ewert et al., 2018) to which our metathalamic probability maps can contribute.

The high-resolution MGB BigBrain maps show the topography of the three subdivisions at nearly cellular resolution, and are interoperable with any reference space used in the neuroimaging community. This way, they can be used to bridge the microscale histology of the metathalamic BigBrain maps with macroscale functional measurements. Evidence from ultrahigh-field-fMRI studies for example shows a mirror-symmetric tonotopic gradient in the ventral MGB (Moerel et al., 2015), which is well reflected by the row-like cell clusters (Winer, 1984; Moerel et al., 2015) that were also detected in the BigBrain (Figure 3B). At the same time, the MGB and its ascending and descending connections seem to be involved in a tinnitus-related network (Rauschecker et al., 2010; Leaver et al., 2011; Caspary and Llano, 2017). Modulation of the ventral MGB is also behaviorally relevant for speech recognition (Mihai et al., 2019) explaining the MGB’s involvement in developmental dyslexia (Díaz et al., 2012). The MGB BigBrain maps may facilitate studies of these larger scale involvements of thalamocortical circuits and local subcortical circuits.

Together with the already published LGB BigBrain maps (Brandstetter et al., 2021), the MGB maps provide a subcortical target space for neuroimaging data integration and comparative histological approaches at the level of specific subdivisions and layers of the metathalamus. Several studies have already used the 20-micron isotropic resolution of the BigBrain dataset for such integrative approaches (Paquola et al., 2020a; Royer et al., 2020) including subcortical structures of the auditory system (Sitek et al., 2019). The BigBrainWarp toolbox (Paquola et al., 2021, preprint) and the EBRAINS VoluBA toolbox for spatial anchoring in the BigBrain space^3^ enable such an integration.

Such a relationship is not only relevant to support MR measurements with the cellular architecture, but also to develop better and more realistic human brain models. The incorporation of cytoarchitectonic parameters has recently led to more biologically valid models of the macaque visual system including cortical areas of different architectural types (Schmidt et al., 2018), as well as models of the human cerebellar granular layer (Florimbi et al., 2021). However, such models usually lack quantitative metathalamic input parameters, forcing them to be estimated indirectly based on other network parameters (Schmidt et al., 2018). Following this line of arguments, the metathalamic maps in the BigBrain can enrich current brain modeling approaches by directly extracting cytoarchitectonic features from the BigBrain (Paquola et al., 2020a; Dickscheid, 2021) at the cellular level (Dickscheid et al., 2019; Behuet et al., 2021). Recent advances in reconstructing the white matter fiber architecture from Nissl-stained glia cells (Schurr and Mezer, 2021) could allow to complement such features with sample specific connectivity data of layers and subdivisions of the metathalamus.

As the BigBrain dataset is continuously expanded by cortical and subcortical cytoarchitectonic parcellations, as well as intracortical surface models (DeKraker et al., 2020; Paquola et al., 2020a; Wagstyl et al., 2020), it provides an increasingly rich resource for such integrative approaches. The here provided high-resolution maps of the MGB contribute to this development.

## Data Availability Statement

The probability maps of the MGB and LGB (Kiwitz et al., 2021a,b) and the MGB BigBrain maps along with the respective reference delineations (Kiwitz et al., 2021c; Schiffer et al., 2021b) are available on EBRAINS: https://ebrains.eu/.

## Ethics Statement

The studies involving human brain tissue from body donors were reviewed and approved by Ethics Committee of the Medical Faculty of the Heinrich Heine University Düsseldorf. The body donors provided written informed consent for the general use of their brain tissue for aims of research and education.

## Author Contributions

KK developed the concept for the manuscript, conducted the cytoarchitectonic brain mapping, validated the quality of the maps and wrote the manuscript. CS designed and implemented the deep-learning based brain mapping tool and created automatic mappings. AB validated the quality of the results of the MGB maps in the BigBrain and contributed LGB specific parts of the manuscript. SB conducted the comparative volumetric analysis of the ten postmortem brains and provided methodological advice. HM provided the calculations for image registration in the BigBrain dataset, calculated the probabilistic maps and integrated them into the Julich-Brain. PM and MO provided critical advice on 3D-visualizations and calculated smoothed volume meshes for the BigBrain maps. KA developed the concept of the study, coordinated the research, and provided neuroanatomical expertise. All authors contributed to the article and approved the submitted version.

## Conflict of Interest

The authors declare that the research was conducted in the absence of any commercial or financial relationships that could be construed as a potential conflict of interest.

## Publisher’s Note

All claims expressed in this article are solely those of the authors and do not necessarily represent those of their affiliated organizations, or those of the publisher, the editors and the reviewers. Any product that may be evaluated in this article, or claim that may be made by its manufacturer, is not guaranteed or endorsed by the publisher.

## Footnotes

1
https://interactive-viewer.apps.hbp.eu/

2
https://ebrains.eu/

3
https://ebrains.eu/service/voluba/

## Supplementary Material

Supplementary Video 1 Zoom into the BigBrain model, left hemisphere, latero-caudal view (cutting plane corresponds to coronal section number 3290, rostral MGB). Subdivisions of the MGB are shown on the right, layers of the LGB are shown on the left.
